# Metabolic control, adherence to the gluten-free diet and quality of life among patients with type 1 diabetes and celiac disease

**DOI:** 10.1186/s13098-023-01167-x

**Published:** 2023-09-28

**Authors:** Ayman A. Al Hayek, Wael M. Al Zahrani, Hamad M. AlAblani, Mohamed A. Al Dawish

**Affiliations:** Department of Endocrinology and Diabetes, Diabetes Treatment Center, Prince Sultan Military Medical City, P.O. Box 7897, Riyadh, 11159 Saudi Arabia

**Keywords:** Metabolic control, Gluten-free diet, Quality of life, Type 1 diabetes, Celiac disease

## Abstract

**Aims:**

In this cross-sectional study, we aimed to evaluate metabolic control, adherence to a gluten-free diet (GFD), and quality of life (QoL) in individuals with type 1 diabetes (T1D) and celiac disease (CD).

**Methods:**

We targeted individuals with T1D and CD at a major tertiary hospital in Saudi Arabia. We gathered retrospective data from medical records and prospectively assessed glycemic control using HbA1c and ambulatory glucose metrics, adherence to a GFD using the Celiac Dietary Adherence Test (CDAT), and QoL using the Celiac Disease Quality of Life survey (CD-QoL).

**Results:**

Forty-eight out of 1095 patients screened (4.38%) were included. Mean age and HbA1c were 21.3 (± 6.6) and 8.3% (± 0.8%). The average time in range% and above range% were 38.5 (range 24–68) and 29.6 (± 7.4). The median hypoglycemic events/month was 8, with a median duration of 80 min. The median overall CDAT and CD-QoL scores were 20.5 and 54. No significant correlations were observed between glucose management indicator (GMI), % in target, and CDAT/CD-QoL scores (all p > 0.05).

**Conclusions:**

No significant effect of GFD on QoL or glycemic control was observed. Further prospective studies are warranted to establish solid evidence of the impact of GFD on individuals with T1D and CD.

**Supplementary Information:**

The online version contains supplementary material available at 10.1186/s13098-023-01167-x.

## Introduction

Type 1 diabetes (T1D), one of the most prevalent chronic disorders in childhood, is characterized by insulin insufficiency resulting from the loss of insulin-producing pancreatic β-cells [1]. Despite the rising prevalence of type 2 diabetes, T1D continues to be the most frequent form of diabetes in children [2]. Symptoms often begin during infancy or adolescence but might manifest later in life [3, 4]. The average life expectancy of individuals with T1D is around 12 years shorter than that of the general population [5]. In Saudi Arabia, the incidence of T1D has increased during the last three decades. Nonetheless, statistics on the incidence and prevalence of T1D remain sparse, and few systematic reviews on the epidemiological data of diabetes in this area have been done [6, 7].

While the causes and risk factors linked with T1D are not entirely understood, the curative and preventive efforts devised so far have failed, with the majority of individuals requiring insulin injections for the entirety of their lives [8]. Current innovations in insulin therapy include the application of insulin pumps (IP), Continuous Glucose Monitoring (CGM), as well as hybrid closed-loop systems [9]. Most individuals with T1D nevertheless experience microvascular and macrovascular complications, even though strict glycemic control may lower the risk of such consequences [9, 10].

Adolescents and younger children with T1D are at an increased risk of developing additional autoimmune disorders, with CD being among the most prevalent co-occurring conditions [11]. The association between T1D and CD is multifaceted and involves serologic, genetic, and clinical aspects. Serologic evidence is detected by identifying specific antibodies, such as anti-endomysial or tissue transglutaminase (tTG) antibodies. Approximately 5-10% of individuals with T1D exhibit this serologic evidence [12, 13], underscoring an underlying immune system dysregulation connecting the two conditions. Moreover, about 5% of T1D individuals are confirmed to have CD through small bowel biopsy [14, 15], often within five years of T1D onset [15], emphasizing the need for vigilant monitoring and screening during early T1D diagnosis.

Specific genes have been implicated in the genetic predisposition to both diseases [16]. For instance, alleles such as DQB1*06:02 and DRB1*04 are considered protective, while haplotypes like HLA-DR3-DQ2 and DR4-DQ8 are linked to an elevated risk for both CD and T1D. This shared genetic susceptibility suggests a common underlying genetic basis for these conditions [17, 18]. In Saudi Arabia, where the prevalence of both CD and T1D is relatively high, the impact of consanguinity, endogamy, and first-cousin marriage on the rise in homozygosity of HLA haplotypes and non-HLA genes has been noted [19, 20]. This may contribute to the increased likelihood of co-occurrence of CD and T1D in this population.

Clinically, the presentation of CD and T1D together can vary. While some individuals may experience gastrointestinal symptoms like food intolerance, avoidance, gastrointestinal pain, and diarrhea, these symptoms are not always evident, particularly in children. Instead, more common preliminary findings include unpredictable glycemic levels, frequent episodes of hypoglycemia, poor glycemic control, and growth failure [21, 22].

The frequency of CD and its potential effect on individuals with T1D necessitates routine CD screening for all children with T1D, as recommended by the American Diabetes Association Professional Practice Committee [23]. For those individuals diagnosed with T1D who subsequently tested positive for CD, the recommended treatment approach is GFD. This dietary intervention should be overseen by a certified dietitian experienced in managing individuals with both T1D and CD. This expertise is essential as gluten-free substitutes often contain higher carbohydrate levels. Therefore, carefully selecting gluten-free products with appropriate carbohydrate content is vital for effective patient management and overall health.

The current evidence highlights the need for further research to bridge the gap in understanding the clinical implications, risk factors, and timing of autoimmune disorders, particularly CD, in the context of T1D among adolescents and younger children. Such investigations can pave the way for improved clinical management and better-informed strategies for this vulnerable population. In the current study, we aimed to evaluate the metabolic control, adherence to a gluten-free diet (GFD), and quality of life (QoL) in individuals with both T1D and CD who sought care at the endocrinology and diabetes center located at Prince Sultan Military Medical City (PSMMC) in Riyadh, Saudi Arabia.

## Methods

### Study design

The PSMMC Research and Ethics Committee approved the research protocol (IRB approval number #E-2030). Before initiating the study assessment, all participants gave oral and written informed permission after being fully briefed on the goals and research methods. Individuals under 18 years were required to provide verbal consent and receive written informed consent from their parents or caregivers. This single-center study was conducted at the Department of Endocrinology and Diabetes, Diabetes Treatment Center, PSMMC, Saudi Arabia. During the first phase, between January and the end of December 2022, we performed a retrospective case review of the medical records of 1,095 individuals with T1D to identify people with dual T1D and confirmed CD.

### Study Population

We included individuals fulfilling the following criteria: (a) aged between 14 and 40 years, (b) who had anti-tTG antibody testing or an endoscopic biopsy to confirm the diagnosis of CD, and (c) who agreed to participate in the study, and signed the informed consent form.

### Study objectives

Our study objectives were to (a) assess the level of glycemic control among study participants based on glycated hemoglobin (HbA1c) and ambulatory glucose profile (AGP) metrics and (b) investigate the correlations between dietary adherence as well as the QoL with glycemic control.

In order to accomplish these objectives, the following data was gathered: socio-demographic and clinical characteristics including age, gender, POCT for HbA1c testing using the cobas b 101 HbA1c analyzer, a point-of-care device manufactured by Roche, treatment modality, T1D duration, duration of proven celiac screening diagnosis, presence of thyroid disease, calculated total daily dose of insulin, and AGP metrics [i.e., glucose variability (GV%), time in range (TIR), time above range (TAR), time below range (TBR)], and average duration of hypoglycemic events for the last 90 days ambulatory glucose profile (AGP) metrics collected using the software to generate patient reports by (LibreView®; Abbott Diabetes Care, Inc.). During the second phase, which ran from January 2023 to March 2023, the study questionnaires were prospectively evaluated in addition to acquiring HbA1c and AGP data.

### Gluten-free diet (GFD) adherence

The extent to which the participants adhered to a GFD was evaluated during routine individual visits using the Celiac Dietary Adherence Test (CDAT) [24]. The CDAT is a self-administered, seven-item survey instrument that has been validated. It contains two queries regarding persistent symptoms (i.e., headaches as well as low energy) and five queries regarding attitudes and behaviors linked to gluten exposure (including a query regarding how often people intentionally consume gluten). Overall scores range between 7 and 35, with higher values indicating poorer adherence to the GFD. Poor adherence is indicated by total scores of more than 13 [24]. The full instruction for using the CDAT is presented in Appendix S1.

### Quality of Life Assessment (CD-QoL)

We used the Celiac Disease Quality of Life survey (CD-QoL), a 20-item self-report CD-specific QoL measure using a 5-point Likert scale to categorize the replies, with 1 denoting “not at all,” 2 “slightly,” 3 “moderately,” 4 “quite a bit,” and 5 denoting “a great deal.” The survey evaluates four subdomains of QoL (dysphoria, limitations, health concerns, and inadequate treatment) and provides an overall QoL score. Scores range between 0 and 100, with higher scores indicating a better life quality [25]. Appendix S2 shows the instructions for using the CD-QoL survey.

### Statistical analysis

Statistical analysis was done using IBM SPSS® Statistics version 26 (IBM® Corp., Armonk, NY, USA). Numerical data were expressed as mean and standard deviation or median and range as appropriate. Qualitative data were expressed as frequency and percentage. Quantitative data were tested for normality using the Kolmogorov-Smirnov and Shapiro-Wilk tests. Correlation between quantitative variables was done using Spearman’s rho correlation. A p-value < 0.05 was considered significant.

## Results

### Socio-demographic characteristics

Forty-eight participants (25 male and 23 female) with T1D and CD were identified, with a mean age of 21.3 (± 6.6) years and a mean HbA1c of 8.3% (± 0.8%). The duration of diabetes ranged from 3 to 21 years, with a median of 8.5 years. The mean duration of positive celiac screening was 6.5 years. Thirty-eight participants (79.2%) were using multiple daily injections (MDI), and 10 participants (20.8%) were using an insulin pump (IP). Thyroid diseases were documented in 8.9%. The demographic data of the study population are shown in ​Table 1.

**Table 1:** Demographic data among the study population

		N = 48	
Age (year)	Mean (SD)	21.3	6.6
Weight (Kg)	Mean (SD)	66.1	10.9
Height (cm)	Mean (SD)	162.4	8.2
Body mass index	Mean (SD)	24.9	2.2
Duration of diabetes (years)	Median (range)	8.5	3–21
Duration of positive celiac screening	Mean (SD)	6.5	3
HbA1c (%)	Mean (SD)	8.3	0.8
Daily dose of insulin– IU	Mean (SD)	1.2	0.3

IU = International units; SD = Standard deviation; HbA1c: glycated hemoglobin

### Level of glycemic control

The mean Glucose Management Indicator (GMI) among the studied population was 8.5 (± 0.8). Meanwhile, the mean value of % time sensor is active, and GV% were 41.7 (± 5.1) and 89.4 (± 5.7). TIR% ranged from 24 to 68, with a median value of 38.5, while the mean TAR% was 29.6 (± 7.4), as shown in Table 2. The mean daily dose of insulin was 1.2 IU (± 0.3). The median values for level 1 hypoglycemia (54–69 mg/dL), level 2 hypoglycemia (< 54 mg/dL), and level 2 hyperglycemia (> 250 mg/dL) were 4 (range 1–8), 1 (range 0–4), and 9 (range 3–30). The median hypoglycemic events/per month was 8. The median duration of hypoglycemic events was 80 min (Table 2).

**Table 2:** Level of Glycemic control among the study population

		N = 48		
Average Glucose		Mean (SD)	207.3	28.4
Glycemic variability (%CV)		Mean (SD)	41.7	5.1
GMI (estimated HbA1C%)		Mean (SD)	8.5	0.8
TIR (Glucose 70–180 mg/dL)		Median (range)	38.5	24–68
TBR; level 1 hypoglycemia (Glucose 54–69 mg/dL)		Median (range)	4	1–8
TBR; level 2 hypoglycemia (Glucose < 54 mg/dL)		Median (range)	1	0–4
TAR; level 1 hyperglycemia (Glucose 181–250 mg/dl)		Mean (SD)	29.6	7.4
TAR; level 2 hyperglycemia (Glucose > 250 mg/dL)		Median (range)	9	3–30
Hypoglycemic events/month		Median (range)	8	3–16
% Time Sensor is Active		Mean (SD)	89.4	5.7
Duration of hypoglycemic events (minutes)		Median (range)	80	14–141

TAR = time above range; TBR = time below range; TIR = time in range; SD = Standard deviation; HbA1c: glycated hemoglobin; GMI = Glucose Management Indicator

### CDAT and CD-QoL

The median CDAT total score among the studied population was 20.5 (15–26). The median values for CD-QoL score, dysphoria, limitation, health concerns, and inadequate treatment domains were 54 (43–62), 9 (7–14), 26 (15–30), 13 (8–18), and 6 (3–8).

### Correlation analysis

**Fig. 1:**
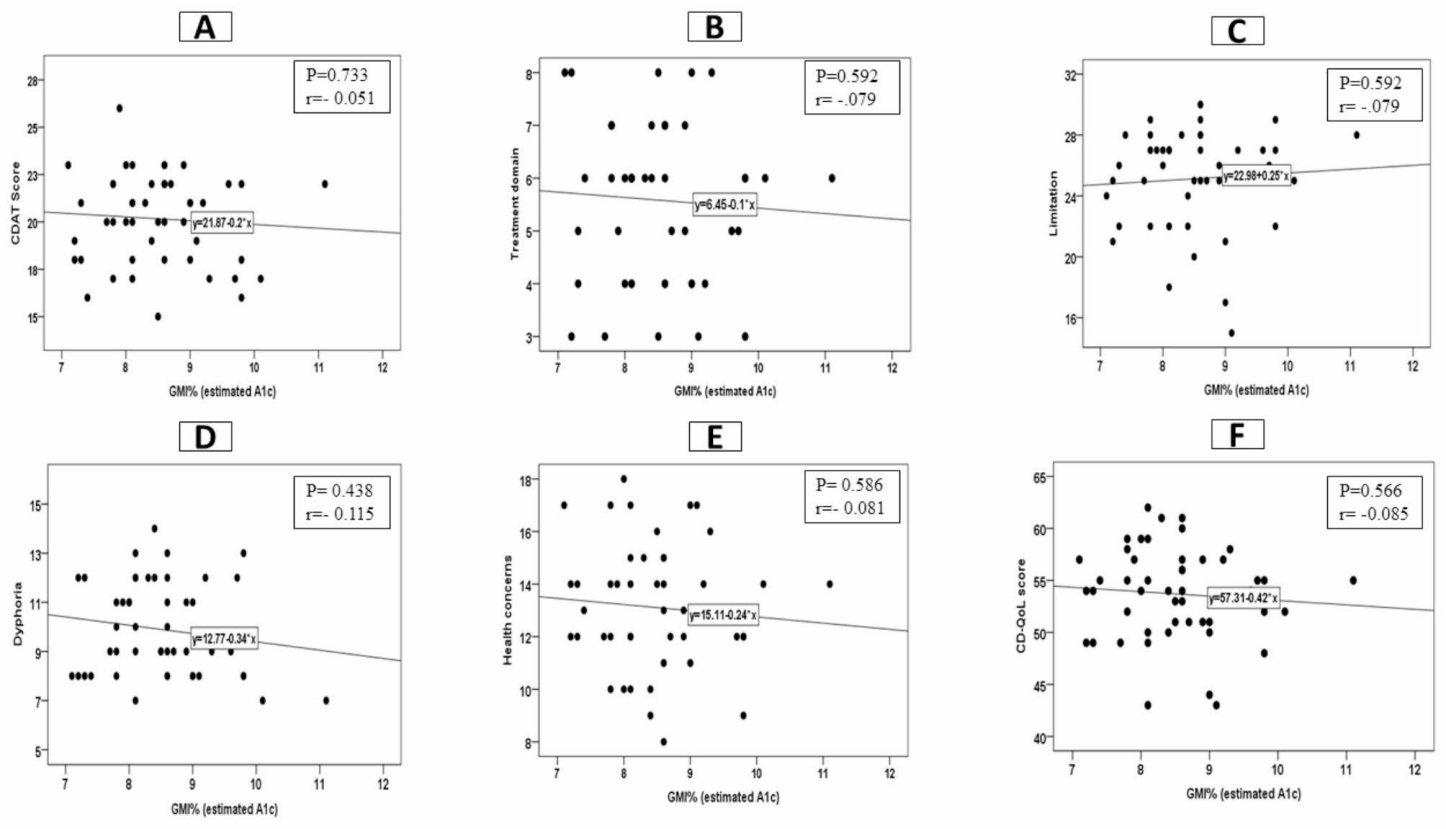
Correlation between CDAT as well as CD-QoL and GMI% (**a**) CDAT score, (**b**) inadequate treatment domain, (**c**) limitation, (**d**) dysphoria, (**e**) health concerns, and (**f**) CD-QoL score. *CDAT = Celiac Dietary Adherence test; CD-QoL = Celiac Disease Quality of Life survey; GMI%= Glucose management indicator*

**Fig. 2:**
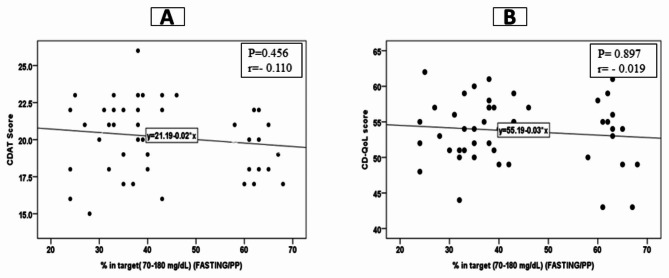
Correlation between different CDAT as well as CD-QoL and % in target (Glucose 70–180 mg/dL)

We observed insignificant correlations between GMI% as well as % TIR (glucose 70–180 mg/dL) and CDAT total score and CD-QoL scale, with all p < 0.05, Fig. 1 and Fig. 2. We observed a significant weak correlation between the duration of positive celiac screening and CD-QoL score (r = 0.315, p = 0.03), but not with subscales and CDAT score, Table 3.

**Table 3:** Correlations of participants’ duration of positive celiac screening and CDAT as well as CD-QoL.

		CDAT	Dysphoria	Limitation	Health concerns	Treatment	CD-QoL Score
Duration of positive celiac screening	r	0.073	0.25	0.145	0.169	0.031	0.315
	p	0.623	0.087	0.325	0.25	0.832	0.029
	N	48	48	48	48	48	48

CDAT = Celiac Dietary Adherence test; CD-QOL = Celiac Disease Quality of Life survey

## Discussion

Our study investigated metabolic control, adherence to GFD, and QoL among individuals with T1D and CD in our center. Out of the 1,095 patients with T1D attending our Diabetes Treatment Center, 48 (4.38%) met the inclusion criteria. Of them, 23 (47.9%) were adults (> 18 years), 28 (58.3%) had DM for < 10 years, and 40 (83.3%) had a positive celiac screening for < 10 years. All people with T1D use the CGM for glucose monitoring in our center. In our study, we observed uncontrolled diabetes among participants indicated by HbA1c values and CGM metrics [i.e., GV%, %TIR, % TAR, TBR, and average duration of hypoglycemic events). Moreover, the CDAT total score ranged between 15 and 26, with a median of 20.5, indicating “*further support to help manage a gluten-free diet is likely beneficial*.” Moreover, the CD-QoL total score was 54, showing moderate Qol, given that a score of 40 or less indicates poor Qol, whereas a score of 60 or more indicates good Qol [25].

Uncertain is whether a GFD helps glycemic control in people with diabetes and CD. Few studies assessed how a strict GFD affects people with T1D and a silent CD [26–28]. Nevertheless, there was no change in insulin requirements among those who adopted GFD [29]; a higher body mass index and enhanced bone health were documented [27, 28]. Some trials have shown either an increase in postprandial glycemic excursions [12, 30], a decrease in severe hypoglycemia episodes [31], or no significant changes [32]. GFD did not seem to affect the emergence of long-term diabetic complications in Creanza et al. [10].

According to a recent meta-analysis, children diagnosed with T1D and CD did not impact their body mass index or HA1c levels following a GFD. This might be ascribed to malabsorption in the absence of serum antibodies since HA1c levels were often lower in years preceding CD diagnosis in the same individuals [33]. Specific characteristics of malabsorption may be due to the existence of antibodies in the duodenal mucosa prior to their appearance in the blood, as shown by studies [34, 35]. On the introduction of a GFD, an increase in HA1C levels was documented; however, this may be due to the restored small intestine mucosa and improved absorption capacity. Nonetheless, at least a year of follow-up showed this was insignificant [28, 36, 37].

Few studies have examined how a GFD affects health-related QoL (HRQoL) in people with T1D and CD, and the findings have been inconsistent. In a recent study [38], out of the 2387 individuals with T1D screened, 82 had biopsy-confirmed CD. Symptom onset and an increase in HbA1c were associated with a decline in HRQoL and self-perceived wellness over the period of 12 months, but the adoption of a GFD had no significant effect on these outcomes. The authors concluded that such individuals might switch to a GFD without negatively affecting their QoL. The findings reported by Weimanare et al. [38] suggest that following a GFD was linked to modest improvements in HRQoL over a year, and there were no significant differences in HRQoL seen between the 2 dietary groups. The extent to which individuals with diabetes already possess skills in self-management and self-efficacy may be relevant to such findings. This is consistent with a cross-sectional study that also failed to show a significant correlation between GFD adherence and HRQoL in individuals with T1D and CD [39].

Similar results were shown in a case-control study including people with T1D and recently diagnosed CD; following a GFD for 12 months had no effect on HRQoL [40]. In contrast, a study found that adherence to a GFD was associated with improved HRQoL in adults [41]. In another case-control study, adherent youth with T1D and CD reported considerably higher general and diabetes-specific HRQoL than nonadherent participants [42]. Nevertheless, except for Leeds et al. [40], these observational studies were either cross-sectional or retrospective, did not rigorously investigate dietary adherence, and included an untreated control group. Additionally, no differentiation of individuals was made in these studies based on CD symptoms, and the vast majority of tested individuals have no symptoms. Previous literature documented that total QoL scores were linked adversely with DM duration; QoL scores decreased as DM duration increased [43]. This is in line with our finding; the median score of the “health concerns” subdomain was higher in those with DM duration ≥ 10 years than > 10 years (p = 0.024).

Individuals with T1D are more likely to experience thyroid autoimmunity, one aspect of the multifaceted interdependent interplay between thyroid disorders and DM. About 8.9% of the study participants had thyroid diseases; this percentage is higher than those reported in previous studies [44–46]. Children with T1D should have their thyroid-stimulating hormone (TSH) levels monitored on a regular basis [23]. Common in this group, autoimmune thyroiditis may alter prognosis. The mean GMI% (estimated A1c) was higher in those with thyroid disease (8.9 vs. 8.3, p = 0.027), with a higher likelihood of experiencing hypoglycemia (glucose < 70 mg/dl).

Similarly, in Orzan et al. [47], the link between thyroid autoimmunity and glycemic imbalance was a key finding. Higher HbA1c levels were seen in T1D individuals with thyroid autoimmunity, consistent with previous literature [48]. A previous study in Saudi Arabia documented that the prevalence of auto thyroid antibodies among Saudi children with T1D was high, and these autoantibodies may indirectly impact glucose regulation. Subclinical thyroiditis is fairly uncommon in people with T1D; hence, it is advised to do a thyroid profile examination, including testing for thyroid auto-antibodies, on a periodic basis [49]. Thus, TSH should be measured routinely in all children and adolescents with T1D as part of their autoimmune thyroiditis monitoring, with fT4 (or total T4) tested reflexively if TSH is abnormal.

### Study limitations and recommendations for future directions

The present study possesses some limitations that warrant consideration in future research. The primary constraint pertains to the limited generalizability of the findings, as the study focused on a single center and a relatively uniform sample. This restriction makes it challenging to extrapolate the results to a broader population. Furthermore, reliance on self-reported data introduces the potential for recall bias, impacting the accuracy of reported behaviors. Additionally, the small sample size may limit the study’s statistical power, and the likelihood of underdiagnosing glycemic issues further complicates the interpretation of results. The study also did not account for crucial variables such as personality traits, parental influences, and the presence of diagnosable eating disorders.

To address these limitations and contribute to a more comprehensive understanding of adherence and glycemic control, future research directions should be considered. Longitudinal studies can provide insights into how these factors evolve over time, offering a more dynamic perspective. Moreover, expanding the participant demographics to include a more diverse population would enhance the generalizability of findings. Objective measurement tools can reduce the impact of recall bias and provide real-time data on glycemic control. Psychosocial assessments, including personality traits and parental influences, should be integrated into research to identify how these factors interplay with adherence and glycemic control. Moreover, researchers should develop interventions tailored to the identified limitations and factors, including educational programs, counseling services, or technology-based solutions. Lastly, systematic screening for underdiagnosis of glycemic issues within healthcare settings can ensure that individuals receive appropriate care and support.

## Conclusion

In conclusion, none of the favorable or adverse effects of a GFD on QoL or glycemic control were established in the current study. More observational and controlled trials are warranted to establish solid evidence of the effects of adherence to GFD in children with T1D and CD and its actual impact on their QoL.

### Electronic supplementary material

Below is the link to the electronic supplementary material.

Appendix S1: Celiac Dietary Adherence Test (CDAT)

Appendix S2: Instructions for Scoring the Celiac Disease Quality of Life survey (CD-QOL)

## Data Availability

The datasets generated during and/or analyzed during the current study are available from the corresponding author upon reasonable request.

## Abbreviations

AGP: Ambulatory Glucose Profile
CD: Celiac Disease
CDAT: Celiac Dietary Adherence Test
CD-QoL: Celiac Disease Quality of Life survey
CGM: Continuous Glucose Monitoring
GFD: Gluten-Free Diet
GMI: Glucose Management Indicator
HRQoL: Health-Related QoL
IP: Insulin Pumps
MDI: Multiple Daily Injections
QoL: Quality Of Life
TAR: Time Above Range
TBR: Time Below Range
T1D: Type 1 Diabetes
TIR: Time In Range
TSH: Thyroid-Stimulating Hormone

## Declarations

### Medical writing and Editorial Assistance

The authors are thankful for the editorial and writing assistance provided by Dr. Mahmoud A. Ebada and Dr. Omar M. Hussein of RAY-CRO during the preparation of this article, funded by Roche Diagnostics Saudi Arabia.

### Submission declaration

All authors agreed to publish the manuscript. This manuscript has not been published previously and is not under consideration for publication elsewhere. All authors are aware that if the study gets accepted, it will not be published elsewhere in the same form, in English or any other language, even electronically, without the written consent of the copyright-holder.
