# Supplementary material for: Metabolic control, adherence to the gluten-free diet and quality of life among patients with type 1 diabetes and celiac disease
Source: Diabetol Metab Syndr. 2023 Sep 28;15:189. doi: 10.1186/s13098-023-01167-x (PMC10536816; doi:10.1186/s13098-023-01167-x)
Supplement: Supplementary file 1 — Appendix S1: Celiac Dietary Adherence Test (CDAT) [file 13098_2023_1167_MOESM1_ESM.pdf]

Coeliac Dietary Adherence Test (CDAT)

Question	Score				
	**1**	**2**	**3**	**4**	**5**
Have you been bothered by low energy level during the past 4 weeks?	None of the time	A little of the time	Some of the time	Most of the time	All of the time
Have you been bothered by headaches during the past 4 weeks?	None of the time	A little of the time	Some of the time	Most of the time	All of the time
I am able to follow a GFD when dining outside my home	Strongly agree	Somewhat agree	Neither agree nor disagree	Somewhat disagree	Strongly disagree
Before I do something I carefully consider the consequences	Strongly agree	Somewhat agree	Neither agree nor disagree	Somewhat disagree	Strongly disagree
I do not consider myself a failure	Strongly agree	Somewhat agree	Neither agree nor disagree	Somewhat disagree	Strongly disagree
How important to your health are accidental gluten exposures?	Very important	Somewhat important	Neutral/ unsure	A little important	Not at all important
Over the past 4 weeks, how many times have you eaten foods containing gluten on purpose?	0 (never)	1-2	3-5	6-10	> 10

Score between 7 and 12 – This suggests your gluten free diet is likely being well managed.
Score between 13 and 17 – This suggests you could benefit from some extra support on following a gluten free diet
Score 18 and 35 – This suggests further support to help manage a gluten free diet is likely beneficial
