# Supplementary material for: Metabolic control, adherence to the gluten-free diet and quality of life among patients with type 1 diabetes and celiac disease
Source: Diabetol Metab Syndr. 2023 Sep 28;15:189. doi: 10.1186/s13098-023-01167-x (PMC10536816; doi:10.1186/s13098-023-01167-x)
Supplement: Supplementary file 2 — Appendix S2: Instructions for Scoring the Celiac Disease Quality of Life survey (CD-QOL) [file 13098_2023_1167_MOESM2_ESM.pdf]

# Computing CD-QOL Scores

*Summarized by J.B. Hu and Carolyn Morris*

CD-QOL is a survey instrument first developed in 2009 to assess health related quality of life [QOL] of patients with celiac disease [CD]. The instrument was co-developed by Spencer Dorn, MD, Douglas A. Drossman, MD, and Peter Green, MD in the United States.

The original U.S. version of the CD-QOL contains 20 question items relating to symptoms of celiac disease. Patients' answers to each question are recorded in Likert-style ratings labeled 1 through 5, where 1=Not at all, 2=Slightly, 3=Moderately, 4=Quite a bit, and 5=A great deal.

In data analysis, patients' answers are transformed and then computed in order to obtain an overall score and four subscores. The table below lists names [beginning with TOT_] of the overall raw score and 4 subscores, their component items in the survey, and acronyms [beginning with CDQOL_] of 5 final 100-point scores:

| Group | Raw scores [total # of component items] | Sequential order of component items in the CD-QOL survey | Final 100-point score names |
|---|---|---|---|
| 1 | TOT-**OV** for Overall [20] | QOL**1** through QOL**20** [All 20 items in the survey] | CDQOL_**OV** |
| 2 | TOT-**DY** for Dysphoria [4] | QOL**10**, QOL**11**, QOL**12**, QOL**13** | CDQOL_**DY** |
| 3 | TOT-**LM** for Limitations [9] | QOL**1**, QOL**5**, QOL**6**, QOL**7**, QOL**14**, QOL**15**, QOL**16**,QOL**17**,QOL**19** | CDQOL_**LM** |
| 4 | TOT-**HC** for Health Concerns [5] | QOL**2**,QOL**3**,QOL**4**,QOL**18**,QOL**20** | CDQOL_**HC** |
| 5 | TOT-**IT** for Inadequate Treatment [2] | QOL**8**,QOL**9** | CDQOL_**IT** |

Three steps are needed to get the final computed scores:

**Step 1**: transform patients' raw answers by reverse coding all ratings to make

Not at all = 5,
Slightly = 4,
Moderately = 3,
Quite a bit = 2, and
A great deal = 1;

**Step 2**: calculate an overall raw score and four raw subscores by totaling all reverse coded ratings of all component items in each of the 5 groups shown in the table above. For example, TOT-DY (Dysphoria) is obtained by adding up 4 reverse coded ratings of QOL10, QOL11, QOL12, and QOL13 in Group 2; and

**Step 3**: compute final 100-point scores using formulas below:

| Group | Final 100-point Score Names | Computation Formula | Explanation |
|---|---|---|---|
| 1 | CDQOL_OV = | ((TOT_OV - 20)/(80))*100 | - denotes subtraction<br>/ indicates division<br>* means multiplication |
| 2 | CDQOL_DY = | ((TOT_DY - 4)/(16))*100 | |
| 3 | CDQOL_LM = | ((TOT_LM - 9)/(36))*100 | |
| 4 | CDQOL_HC = | ((TOT_HC - 5)/(20))*100 | |
| 5 | CDQOL_IT = | ((TOT_IT -2)/(8))*100 | |

**Note:**

Final scores should range from 0 to 100; no final score should exceed 100.
Higher scores may mean higher degree of QOL *and* less degree of CD symptoms and their impact.

# CD-QOL Survey

**Please think about your life over the past month (30 days),** and look at the statements below. Each statement has five possible responses. **For each statement, please fill in one box in each row that best describes your feelings.**

| | Not at all 1 | Slightly 2 | Moderately 3 | Quite a bit 4 | A great deal 5 |
|---|---|---|---|---|---|
| 1 I feel limited by this disease …… | ☐ | ☐ | ☐ | ☐ | ☐ |
| 2 I feel worried that I will suffer from this disease …… | ☐ | ☐ | ☐ | ☐ | ☐ |
| 3 I feel concerned that this disease will cause other health problems …… | ☐ | ☐ | ☐ | ☐ | ☐ |
| 4 I feel worried about my increased risk of cancer from this disease …… | ☐ | ☐ | ☐ | ☐ | ☐ |
| 5 I feel socially stigmatized for having this disease …… | ☐ | ☐ | ☐ | ☐ | ☐ |
| 6 I feel like I'm limited in eating meals with coworkers …… | ☐ | ☐ | ☐ | ☐ | ☐ |
| 7 I feel like I am not able to have special foods like birthday cake and pizza …… | ☐ | ☐ | ☐ | ☐ | ☐ |
| 8 I feel that the diet is NOT sufficient treatment for my disease …… | ☐ | ☐ | ☐ | ☐ | ☐ |
| 9 I feel that there are not enough choices for treatment …… | ☐ | ☐ | ☐ | ☐ | ☐ |
| 10 I feel depressed because of my disease …… | ☐ | ☐ | ☐ | ☐ | ☐ |
| 11 I feel frightened by having this disease …… | ☐ | ☐ | ☐ | ☐ | ☐ |
| 12 I feel like I don't know enough about the disease …… | ☐ | ☐ | ☐ | ☐ | ☐ |
| 13 I feel overwhelmed about having this disease …… | ☐ | ☐ | ☐ | ☐ | ☐ |
| 14 I have trouble socializing because of my disease …… | ☐ | ☐ | ☐ | ☐ | ☐ |
| 15 I find it difficult to travel or take long trips because of my disease …… | ☐ | ☐ | ☐ | ☐ | ☐ |
| 16 I feel like I cannot live a normal life because of my disease …… | ☐ | ☐ | ☐ | ☐ | ☐ |
| 17 I feel afraid to eat out because my food may be contaminated …… | ☐ | ☐ | ☐ | ☐ | ☐ |
| 18 I feel worried about the increased risk of one of my family members having celiac disease. | ☐ | ☐ | ☐ | ☐ | ☐ |
| 19 I feel like I think about food all the time …… | ☐ | ☐ | ☐ | ☐ | ☐ |
| 20 I feel concerned that my long term health will be affected …… | ☐ | ☐ | ☐ | ☐ | ☐ |
| | 1 Not at all | 2 Slightly | 3 Moderately | 4 Quite a bit | 5 A great deal |

21. How would you rate your quality of life related to your illness? *[Please check one]*

☐ 5 Excellent ☐ 4 Very Good ☐ 3 Good ☐ 2 Fair ☐ 1 Poor

*THANK YOU FOR FILLING OUT THIS SURVEY!*
